# Influence of acute consumption of caffeine vs. placebo over Bia-derived measurements of body composition: a randomized, double-blind, crossover design

**DOI:** 10.1186/s12970-018-0211-5

**Published:** 2018-02-13

**Authors:** Cassie M. Williamson, Brett S. Nickerson, Emily E. Bechke, Cherilyn N. McLester, Brian M. Kliszczewicz

**Affiliations:** 1 Department of Exercise Science & Sport Management, Kennesaw State University, Kennesaw, GA USA; 2 Department of Professional Programs, Texas A&M International University, Laredo, TX USA

**Keywords:** Total body water, Body fat percent, Pretesting criteria, Intracellular water, Extracellular water

## Abstract

**Background:**

Bioelectrical impedance analysis (BIA) is often used to estimate total body water (TBW), intracellular body water (ICW), extracellular body water (ECW), and body fat percentage (BF%). A common restriction for BIA analysis is abstinence from caffeine 12-h prior to testing. However, research has yet to determine whether the consumption of caffeine influences BIA testing results. The purpose of this study was to determine if the consumption of caffeine influences BIA-derived BF% and body water values in habitual caffeine users.

**Methods:**

Twenty apparently healthy males (26.6 ± 4.1 years) identified as habitual caffeine consumers (≥ one 95 mg serving per day ≥ four days per week) participated in this study. Participants came to the lab on three occasions, the first visit serving as the control (CON) with no supplementation. The remaining two visits were performed in a randomized double-blind, cross-over fashion. Participants consumed 200 mg of dextrose (PLA) or caffeine (CAF) in capsule form. During each visit, seven multi-frequency BIA measurements were conducted before (PRE) and after (15-min, 30-min, 45-min, 60-min, 75-min, 90-min) consumption.

**Results:**

Repeated measures ANOVA revealed BF% for CAF was lower than the CON and PLA conditions at PRE and 15-min (*p* < 0.001, *p* = 0.004), but not statistically significant for the remaining time points (i.e., 30-, 45-, 60-, 75-, and 90-min). However, the effect size (ES) of the BF% differences were trivial. The CON, PLA, and CAF conditions had higher PRE ICW values than their associated post time points (i.e., 15-, 30-, 45-, 60-, 75-, and 90-min). Similar to BF%, ES of the mean differences for ICW were trivial. No other differences were observed.

**Conclusion:**

Caffeine consumption in habitual users produced trivial changes in TBW, ECW, ICW, or BF%. Therefore, the pre-testing guidelines for caffeine consumption may not be necessary in habitual caffeine consumers.

## Background

Body composition is commonly assessed in field, clinical, and research settings due to its ability to analyze the effectiveness of exercise programming for weight loss and athletic performance [1]. Emerging technology now allows a more suitable option for body composition assessment occurring outside of research settings. For instance, bioelectrical impedance analysis (BIA) is commonly used in field settings to assess body fat percentage (BF%) and body water values. This method is highly desirable due to its portability and ease of use [2, 3]. Although BIA requires simple procedures and provides greater accessibility within the field, the technique comes with pre-testing restrictions, such as avoidance of exercise, fasting, and abstaining from caffeine consumption prior to testing [4–7]. Within a research setting, these guidelines are realistic to follow. However, these recommendations are not practical in field applications and are less likely to be followed by participants at health-related institutions.

Previous studies have examined certain pre-testing restrictions, such as exercise avoidance, meal consumption, and heat exposure, to determine if these restrictions have any influence over BIA-derived measurements of body composition. For instance, a previous study examined the influence of meal consumption on BF% and demonstrated that strict fasting is not necessary in clinical settings. The elimination of the strict fasting from pre-testing guidelines increases the opportunity for BIA applications within clinical settings [4]. In addition, a study conducted in our lab examined the influence of exercise at both moderate and high-intensities on BIA-derived BF% and found minimal changes after exercise at both intensities [6]. Furthermore, it has been reported that exercise-induced increases in body temperature and skin blood flow have minimal impact on BIA measurements of body composition [7, 8]. In light of several pre-testing guidelines having minimal influence over BIA measurements, other restrictions have yet to be examined, such as the influence of caffeine consumption on measurements of body composition.

Caffeine, which can be consumed in several forms (e.g. coffee, energy drinks, etc.), is one of the most commonly used supplements worldwide [9, 10]. Caffeine is considered a diuretic during periods of rest which results in a greater volume of fluid loss through urine [10, 11]. For instance, a study conducted by Neuhäuser-Berthold et al. [12] demonstrated a loss in total body water (TBW) of approximately 1.1 kg following the consumption of a 321 mg serving of caffeine in the form of coffee in the morning and afternoon. Moreover, Passmore et al. [13] examined the effects of early day caffeine consumption on urine output in several doses (45, 90, 180, and 360 mg) and found a statistical difference following the highest dose. Caffeine’s role as a diuretic is relatively understood, but it is unclear if caffeine consumption truly influences cellular fluid balance, which could affect the calculation of intracellular (ICW), extracellular (ECW), or TBW when measured by BIA. Therefore, the primary purpose of this study was to determine if the consumption of caffeine influences BIA-derived BF% and body water values in habitual caffeine users. Additionally, heart rate was examined prior to BIA analysis in order to provide insight into the role of caffeine related sympathetic nervous system activity and BIA measurements. It has been demonstrated that habitual caffeine consumers are less sensitive to the effects of caffeine [14], and therefore, we hypothesized that caffeine would demonstrate little to no influence over these markers.

## Methods

### Experimental design

All data collection was performed in the Exercise Physiology Laboratory (EPL) and took place on three separate occasions. All three visits occurred between the hours of 6 AM and 12 PM within a seven-day period with a minimum of 24-h between visits to ensure proper washout period. Participants were required to be well hydrated upon arrival, which was determined by a hand-held refractometer (Mannix, DR-303, Lynbrook, NY) to measure urine-specific gravity (USG). USG measurements ≤1.029 were considered adequate [15]. The first visit served as a control (CON) trial where each participants’ height (cm) was measured using a digital physician’s scale (Tanita WB 3000, Arlington Heights, IL) and their weight, BF%, TBW, ECW, and ICW were determined through BIA analysis via the InBody 770 (InBody USA, Cerritos, CA). After the preliminary measurements were completed (i.e., USG and height) participants were instructed not to void their bladder until the completion of testing. BIA measurements were taken at seven different time points separated by 15-min; after preliminary measurements (PRE), measure two (15-min), measure three (30-min), measure four (45-min), measure five (60-min), measure six (75-min), measure seven (90-min). The second and third visits were conducted in a double-blind, randomized crossover fashion between 200 mg of caffeine anhydrous (CAF) and 200 mg of dextrose placebo (PLA) encapsulated in green, non-translucent, size zero gelatin capsules (PurecapsUSA). The capsule was consumed with 8 fluid ounces of water immediately following the PRE measurement. The identity of the contents within the capsules were not revealed until all data were collected and statistical analyses were completed.

### Participants

The University Institutional Review Board approved all testing protocols and procedures of this study and followed the rules and guidelines of the declaration of Helsinki [16]. A total of 20 physically active males (age = 26.6 ± 4.1 years; weight = 81.3 ± 9.8 kg) participated in this study. Inclusion criteria required that participants were habitual caffeine consumers, which was defined as those who consumed a minimum of one serving per day (95 mg) at least four days per week, and physically active with the minimum requirements based on American College of Sports Medicine guidelines [17]. Upon arrival to the EPL, participants were provided the details of the study and a written informed consent was obtained. Next, participants filled out a demographic and health history questionnaire to determine that the inclusion criteria were met. Those who reported having any cardiovascular, pulmonary, or metabolic diseases were excluded from the study. Additionally, those who were taking any medications and/or supplements that would influence hydration status (e.g., diuretics, creatine) or cardiovascular activity (e.g. stimulants) were excluded from this study. All participants were asked to fast for a minimum of 12-h, abstain from alcohol and exercise for 24-h, and avoid caffeine 12-h prior to all testing sessions.

### Procedures

#### Bioelectrical impedance analysis

The BIA measurements were taken at seven time points (i.e., PRE, 15-min, 30-min, 45-min, 60-min, 75-min, and 90-min) for all three trials. Before BIA measurement, participants were instructed to remove their shoes and socks and the bottoms of the feet and palms of the hands were wiped using tissue that contained 0.9% NaCl. Participants were asked to step onto the BIA device, placing the soles of their feet on the foot electrodes and gripping the hand electrodes. The participant was asked to stand with their arms straight and away from the trunk so they were not touching the body to achieve proper testing posture according to the manufacturer’s guidelines. The InBody 770 device estimates BF%, TBW, ECW, and ICW using six different frequencies: 1 kHz, 5 kHz, 50 kHz, 250 kHz, 500 kHz, and 1000 kHz.

### Heart Rate

Heart rate (HR) was measured and recorded prior to each BIA measurement using a Polar HR monitor (Polar T-31, Bethpage, NY). The HR monitor and chest strap was placed directly against the skin underneath the sternum. The HR monitor was then connected to a polar watch (Polar FTI, Bethpage, NY) to read and record the HR one minute prior to each BIA measurement. In order to collect a resting HR in the standing position while avoiding any changes that may occur as a result of orthostatic challenge, participants were asked to stand for the five minutes before each BIA measurement; the HR of the fifth minute was recorded. The HR monitor was then removed before BIA measurement to avoid any interference with the analysis.

### Statistical analysis

According to a priori analysis, the sample size of the current study was sufficient based on a power of 0.8, alpha level of significance of 0.05, and an effect size of 0.3 [6, 18]. A series of condition (CON, CAF, and PLA) x time (PRE, 15-, 30-, 45-, 60-, 75-, and 90-min) repeated measures analysis of variance (RMANOVA) were performed to compare the differences in BF%, TBW, ECW, and ICW mean values between the different testing conditions and time points. Where violations of the sphericity assumption occurred, the Greenhouse-Geisser correction was used to adjust degrees of freedom. In the event of a significant F-ratio, main effects were further assessed by Bonferonni post-hoc analysis, while simple effects for condition and time were analyzed via separate one-way RMANOVA’s with a Bonferonni correction. Effect size (ES) of the mean differences was determined using Cohen’s d. The magnitude of the ES was determined by Hopkin’s scale [19] as follows: 0–0.2 = trivial, 0.2–0.6 = small, 0.6–1.2 = moderate, 1.2–2.0 = large, > 2.0 = very large. A criterion alpha level of *p* ≤ 0.05 was used to determine statistical significance. All data are reported as mean ± standard deviation. Statistical Software (V. 24.0, SPSS Inc., Chicago, IL) was used for all analyses.

## Results

### Total body water

Mean values for BF%, TBW, ECW, and ICW are displayed in Table 1. There was no statistically significant main effect for condition (F_1.551, 29.465_ = 0.097; *p* = 0.861). Conversely, the main effect for time was statistically significant (F_2.931, 55.692_ = 26.211; *p* < 0.001). However, post hoc analysis revealed no statistically significant pairwise comparisons. In addition, no statistically significant condition × time interaction effect was observed (F_12, 228_ = 1.588; *p* = 0.096).

**Table 1:** ᅟ

Marker	CON	Cohen’s d	PLA	Cohen’s d	CAF	Cohen’s d
BF%						
PRE	16.1 ± 6.4	---	16.0 ± 6.4	---	15.5 ± 6.2^bc^	---
15-MIN	16.4 ± 6.3^a^	0.05	16.5 ± 6.4^a^	0.08	16.0 ± 6.2^abc^	c0.08
30-MIN	16.5 ± 6.4^a^	0.06	16.5 ± 6.3^a^	0.08	16.4 ± 6.3^a^	0.01
45-MIN	16.6 ± 6.4^a^	0.08	16.7 ± 6.3^a^	0.11	16.5 ± 6.3^a^	0.16
60-MIN	16.8 ± 6.3^a^	0.11	16.9 ± 6.4^a^	0.14	16.5 ± 6.4^a^	0.16
75-MIN	16.9 ± 6.2^a^	0.13	17.0 ± 6.3^a^	0.16	16.5 ± 6.3^a^	0.16
90-MIN	16.8 ± 6.2^a^	0.11	17.2 ± 6.5^a^	0.19	16.7 ± 6.4^a^	0.19
TBW						
PRE	109.2 ± 11.9	---	109.0 ± 12.1	---	109.4 ± 11.5	---
15-MIN	108.9 ± 12.0	0.03	108.8 ± 12.3	0.02	109.0 ± 11.5	0.04
30-MIN	108.8 ± 11.9	0.03	108.7 ± 12.3	0.03	108.6 ± 11.7	0.07
45-MIN	108.6 ± 11.8	0.05	108.5 ± 12.2	0.04	108.4 ± 11.4	0.09
60-MIN	108.4 ± 11.9	0.07	108.3 ± 12.4	0.06	108.3 ± 11.3	0.10
75-MIN	108.3 ± 12.1	0.08	108.1 ± 12.3	0.07	108.3 ± 11.5	0.10
90-MIN	108.4 ± 12.0	0.07	107.9 ± 12.4	0.09	108.1 ± 11.6	0.11
ICW						
PRE	69.1 ± 7.6	---	68.9 ± 7.7	---	69.3 ± 7.4	---
15-MIN	68.9 ± 7.6	0.03	68.7 ± 7.8	0.03	69.0 ± 7.4^a^	0.04
30-MIN	68.7 ± 7.5^a^	0.05	68.6 ± 7.8^a^	0.04	68.6 ± 7.5^a^	0.09
45-MIN	68.6 ± 7.5^a^	0.07	68.5 ± 7.8^a^	0.05	68.4 ± 7.3^a^	0.12
60-MIN	68.5 ± 7.5^a^	0.08	68.3 ± 7.8^a^	0.08	68.4 ± 7.2^a^	0.12
75-MIN	68.4 ± 7.7^a^	0.09	68.2 ± 7.7^a^	0.09	68.4 ± 7.4^a^	0.12
90-MIN	68.4 ± 7.6^a^	0.09	68.0 ± 7.8^a^	0.12	68.2 ± 7.4^a^	0.15
ECW						
PRE	40.1 ± 4.4	---	40.1 ± 4.5	---	40.1 ± 4.2	---
15-MIN	40.1 ± 4.5	0.00	40.1 ± 4.5	0.00	40.1 ± 4.2	0.00
30-MIN	40.1 ± 4.5	0.00	40.1 ± 4.6	0.00	40.0 ± 4.3	0.00
45-MIN	40.0 ± 4.4	0.02	40.0 ± 4.5	0.02	39.9 ± 4.2	0.05
60-MIN	40.0 ± 4.4	0.02	39.9 ± 4.6	0.04	40.0 ± 4.2	0.02
75-MIN	39.9 ± 4.5	0.05	39.9 ± 4.6	0.04	40.0 ± 4.2	0.02
90-MIN	40.0 ± 4.5	0.02	39.9 ± 4.7	0.04	39.9 ± 4.2	0.05

Body composition markers are presented as the mean ± SD

^a^Represents significance of difference between time points and PRE value of the condition

^b^Represents significant difference between CON condition

^c^Represents significant difference between PLA condition. Cohen’s d values are relative to the corresponding time point when compared to PRE

### Extracellular water

There was no statistically significant main effect for condition (F_2, 38_ = 0.007; *p* = 0.993). Conversely, the main effect for time was statistically significant (F_6, 114_ = 5.725; *p* < 0.001). However, post hoc analysis revealed no statistically significant pairwise comparisons. Furthermore, no statistically significant condition × time interaction effect was observed (F_12, 228_ = 1.220; *p* = 0.270).

### Intracellular water

There was no statistically significant main effect for condition (F_1.541, 29.271_ = 0.288; *p* = 0.694). Conversely, the main effect for time was statistically significant (F_2.950, 56.054_ = 5.949; *p* < 0.001). Post hoc analysis revealed that baseline (i.e., PRE) ICW values for the CON and PLA condition were statistically significant for all post comparisons (all *p* < 0.05) except the 15-min time point (*p* = 0.092 and 0.468, respectively). In addition, baseline ICW values for CAF condition were statistically significant (all *p* < 0.05) for all post comparison time points (i.e., 15-, 30-, 45-, 60-, 75-, and 90-min). Lastly, no statistically significant condition × time interaction effect was observed (F_5.757, 109..392_ = 1.856; *p* = 0.098).

### Body fat percentage

There was a statistical significant condition × time interaction for BF% (F_12, 228_ = 2.605; *p* = 0.003). Post hoc analysis revealed BF% for CAF was lower than CON and PLA at PRE (*p* = 0.001 and 0.009, ES = 0.10 and 0.08, respectively) and 15-min (*p* = 0.56 and 0.004, ES = 0.05 and 0.08, respectively), but not statistically significant for the remaining time points.

### Heart rate

There was a statistically significant condition × time interaction for HR (F_6.928, 131.634_; *p* < 0.001). Post hoc analysis revealed CON had lower HR values when compared to CAF at PRE (*p* = 0.009). However, HR values for CON were higher than CAF at 30-min (*p* = 0.013). No other statistical significance was observed for HR.

## Discussion

The purpose of this study was to determine if the consumption of caffeine influences BIA-derived BF% and body water values in habitual caffeine users. The primary findings of this study demonstrated that there was a significant interaction for BF%. Analysis revealed that BF% for CAF was lower than the CON and PLA conditions at PRE and 15-min but not statistically significant for the remaining time points (i.e., 30-, 45-, 60-, 75-, and 90-min). Although these mean values were statistically significant at PRE and 15-min, the ES of the mean differences were all trivial. As a result, these finding suggest that the significant BF% differences have minimal practical implications. There were also significant main effects for time when evaluating ICW values in all three conditions. Post hoc analysis revealed that the CON, PLA and CAF conditions had higher PRE ICW values than their associated post time points (i.e., 15-, 30-, 45-, 60-, 75-, and 90-min). However, similar to BF%, the effect sizes of the mean differences were all trivial, which once again demonstrates that these differences have minimal practical implications on changes in ICW. Collectively, these findings are suggestive that current restrictions for pre-testing guidelines involving caffeine and BIA may be too stringent.

BIA technology has advanced and is now capable of using high and low frequencies to estimate ICW and ECW, respectively. After determining these values, the estimation of TBW can be derived, which can then be used to estimate BF% based on the assumed hydration of FFM being 73.72% [20]. This is the first study to identify the impact that caffeine consumption has on BF% and body water when using BIA. Research has previously examined the impact of altering BIA pre-testing guidelines such as avoidance of exercise and meal consumption [4, 5]. However, research has yet to emerge on whether the consumption of caffeine can have an influence on BIA-derived body water and BF%.

During the CON conditions, throughout the 15-min intervals, no observed changes in mean TBW were observed. Very few studies have examined fluid consumption and time course evaluation of BIA. When evaluating the TBW in PLA and CAF conditions, there were no significant changes in value despite consuming 8 fluid ounces of water between the PRE and 15-min time points. Our findings are consistent with those of Gibson et al. [21], who observed no significant changes of TBW when measured every five-minutes over a 30-min time period in both standing and supine positions. Importantly, in the aforementioned study, the participants did not consume any food or drink during the duration of testing, unlike the CAF and PLA conditions of the current study. Conversely, Androutsos et al. [4] examined the effects of consuming 750 mL of water with a meal and observed significant changes in TBW when measured every 30-min for up to 2-h. The results of the current study are likely different due to the consumption of a comparatively small amount of water while in a fasted state. A unique aspect of the current study is that throughout the ingestion period, participants were not allowed to void their bladder, therefore, preventing changes in TBW. This however does not explain the lack of TBW increase with the consumption of 8 fluid ounces of water, given that ample time was provided for absorption into the bloodstream, which can occur within five-minutes [22]. It is possible that the 5 frequencies utilized by the BIA device in the current study are not sensitive to the 8 fluid ounces of water consumption. Therefore, future research should determine if higher frequencies and more sophisticated bioimpedance devices, such as bioimpedance spectroscopy, are more sensitive to small quantities of water absorption.

Similar to TBW, no changes were observed in ECW throughout the 90-min period in any of the conditions. Our findings were consistent with those of a study conducted by [23], in which measurement of ECW via the sodium bromide dilution method showed no changes after consumption of 2.5 mg·kg^− 1^·day^− 1^ of caffeine consumed twice per day for four days. Few studies have examined repeated measurements of ECW over a period of time, therefore the response to fluid and supplement ingestion is relatively unknown. It was an expected observation that the CON trial would elicit little to no change in ECW throughout the time points. Through impedance measurements, ECW is quantified using frequencies under 50 kHz, quantifying fluid outside of the cells. For example, the InBody 770 utilizes multiple low frequencies (i.e., 1 kHz, 5 kHz, and 50 kHz) in order to quantify ECW. A potential rationale for a failure to detect changes in ECW is the rate of extracellular fluid flow (i.e., cardiac output). When compared to ICW, which is relatively constant, ECW is more mobile throughout the body and may lead to interference with small transient changes in volume. Consequently, the measurement of TBW with lower impedance frequencies and higher rate of extracellular flow make it difficult to assess ECW.

There were significant decreases observed in ICW through out the 90-min time period. The observed changes occurred in all three conditions, suggesting that CAF was not the primary cause of the decrease in ICW. Although significant decreases were observed, the effect sizes of the differences were trivial, and therefore have minimal practical implications. The decrease in ICW was unexpected due to the stable conditions observed in TBW and ECW throughout the time period. A likely mechanism involved for the decrease in ICW among all conditions was the prolonged periods of rest. At the beginning of all conditions, HR was elevated, likely due to the activity of walking to the laboratory and acclimating to the new environment. The prolonged rest periods throughout the trial could have contributed to a lack of requirement of blood and fluid into large cellular tissue (i.e., skeletal muscle). An additional reason for the observed findings could be the ionic composition of ICW varies amongst different types of cells [24], and therefore, could be a limiting factor due to the potential inability to directly measure ICW.

A time-dependent increase in BF% was observed over the 90-min period. Importantly, the condition elicited little to no effect over BF%, which is an interesting finding due to the current pre-testing guidelines and recommendations requiring the abstinence of caffeine. Caffeine is known to stimulate sympathetic nervous system activity [25] and is also a known diuretic under resting conditions [11], both of which in theory, should influence homeostatic fluid balance. However, habitual caffeine users may demonstrate less sensitivity to caffeine consumption and therefore, have little to no influence over measurements of fluid compartments [14]. Consequently, future research might seek to determine the influence of caffeine on BIA measurements (e.g., body water) at varying doses as well as in individuals who are not considered habitual caffeine users such as that of the current study sample.

Multi-frequency BIA may not be sensitive enough to detect smaller volumes of fluid added to the system. It was anticipated that 8 fluid ounces of water, which is approximately 5% of average systemic volume (i.e., 5-l) [26], would be detected in either TBW, ECW, or ICW. The absorption rate of water from the gastrointestinal tract typically occurs within five-minutes of consumption [22]. Despite this, the only time dependent change within these variables was an observed decrease in ICW for all groups. This is a surprising result, especially considering that PLA and CAF conditions involved consumption of additional fluid, suggesting a lack of sensitivity in the multi-frequency BIA. However, the magnitudes of the significant differences for ICW values were minimal. A possible rationale, as suggested earlier, may be due to the circulation of fluid in the extracellular component.

## Conclusions

Overall, the findings of this study are suggestive that the presence or absence of caffeine prior to multi-frequency BIA provides little influence on BF%, TBW, ECW, or ICW in habitual users. An additional finding of this study showed that the consumption of 8 fluid ounces of water immediately before testing and for up to 90-min also showed no differences in any marker provided by BIA. Lastly, it would appear that acute activity status (e.g., prolonged periods of seated positions) has a greater albeit minimal influence over BF% measurements through multi-frequency BIA. Therefore, practitioners in the field may not benefit from adhering to the caffeine restriction in habitual caffeine users for multi-frequency BIA.

## Abbreviations

BF%: Body Fat Percentage
BIA: Bioelectrical Impedance Analysis
CAF: Caffeine Trial
CON: Control Trial
ECW: Extracellular Water
FFM: Fat Free Mass
HR: Heart Rate
ICW: Intracellular Water
PLA: Placebo Trial
TBW: Total Body Water
USG: Urine Specific Gravity
